# Relevance of HLA-DQB1*02 Allele in the Genetic Predisposition of Children with Celiac Disease: Additional Cues from a Meta-Analysis

**DOI:** 10.3390/medicina55050190

**Published:** 2019-05-22

**Authors:** Cristina Capittini, Annalisa De Silvestri, Chiara Rebuffi, Carmine Tinelli, Dimitri Poddighe

**Affiliations:** 1 Scientific Direction, Clinical Epidemiology and Biometric Unit, Fondazione IRCCS Policlinico San Matteo, 27100 Pavia, Italy; C.Capittini@smatteo.pv.it (C.C.); a.desilvestri@smatteo.pv.it (A.D.S.); ctinelli@smatteo.pv.it (C.T.); 2 Grant Office and Scientific Documentation Center, Fondazione IRCCS Policlinico San Matteo, 27100 Pavia, Italy; C.Rebuffi@smatteo.pv.it; 3 Department of Medicine, Nazarbayev University School of Medicine, Nur-Sultan City 010000, Kazakhstan

**Keywords:** celiac disease, children, HLA-DQB1*02, screening, first-degree relatives

## Abstract

*Background and Objectives:* Celiac disease (CD) is a multifactorial immune-mediated disorder, triggered by the ingestion of gluten in genetically-predisposed subjects carrying MHC-DQ2 and -DQ8 heterodimers, which are encoded by four HLA-DQ allelic variants, overall. This meta-analysis aims at providing further epidemiological support to the predominant relevance of one specific allele, namely HLA-DQB1*02, in the predisposition and genetic risk of CD. *Materials and Methods:* We performed a search of MEDLINE/PubMed, Embase, Web of Science, and Scopus, retrieving all publications (case–control study, cross-sectional, and retrospective cohort study) on the association between HLA class II polymorphisms and first-degree relatives (FDRs) of children with CD. After a critical reading of the articles, two investigators independently performed data extraction according to the following inclusion criteria: HLA class II genes, any DQ and DR molecules, and CD diagnosed following the current clinical guidelines. A third participant was consulted for discussion to reach an agreement concerning discrepancies. *Results:* Our search strategy selected 14 studies as being eligible for inclusion, and those were submitted for data extraction and analysis. These studies were published between 1999 and 2016 and, collectively, enrolled 3063 FDRs. Positive and negative likelihood ratios (LR+ and LR−, respectively) for CD diagnosis, according to the presence of the HLA-DQ genotype coding a complete MHC-DQ2 and/or MHC-DQ8 molecules, were 1.449 (CI 1.279–1.642) and 0.187 (CI 0.096–0.362), respectively. If only the isolated presence of HLA-DQB1*02 allele is considered, the pooled estimation of LR+ was 1.659 (CI 1.302–2.155) and, importantly, the LR− still showed a very good discriminatory power of 0.195 (CI 0.068–0.558). *Conclusions:* Through our differential meta-analysis, comparing the presence of the genotype coding the full MHC-DQ2 and/or DQ8 molecules with the isolated presence of HLA-DQB1*02 allelic variant, we found that the LR− of the latter analysis maintained the same value. This observation, along with previous evidences, might be useful to consider potential cost-effective widened screening strategies for CD in children.

## 1. Introduction

Celiac disease (CD) is a multifactorial immune-mediated disorder, triggered by the ingestion of gluten and other gluten-related proteins in genetically predisposed subjects. Importantly, the HLA-DQ alleles, coding α and β chains of the MHC-DQ2 and -DQ8 heterodimers, have been shown to be a necessary, but not sufficient, immunogenetic background for the development of CD. These HLA-DQ haplotypes have been estimated to contribute up to 25%–40% of the genetic risk for CD and have been reported to be present in around 35–40% of the general population in North America and Europe, where the prevalence of CD is close to 1% and, probably, even more if only the pediatric population is considered [1,2].

In particular, children are a vulnerable population with respect to the complications and long-term consequences of untreated CD, taking into account also their longer life-expectancy. Moreover, in addition to gastrointestinal symptoms, a considerable number of patients with CD present extra-gastrointestinal manifestations only (leading to under-diagnosis and/or significant diagnostic delays), and some patients may be completely asymptomatic, although they often report a subjective improvement after starting a gluten-free diet. Long-term complications of untreated CD are plausible, but there are still few studies addressing this specific issue [2,3,4,5,6].

All these epidemiological and clinical aspects have stimulated the scientific debate about the possibility to implement a wider screening strategy to identify CD patients, especially in children. Indeed, the screening approach by active case-finding, limited to the first-degree relatives (FDRs) of CD patients and children affected with other autoimmune diseases or chromosomal aberrations (known to be statistically associated with CD), was only partially effective as most asymptomatic or mildly symptomatic patients have no clear risk factors and, thus, cannot be detected [7,8]. However, extending the serological screening to all children and repeating it at several ages in childhood, is not a sustainable approach and, therefore, alternative strategies must be sought.

**Figure 1:**
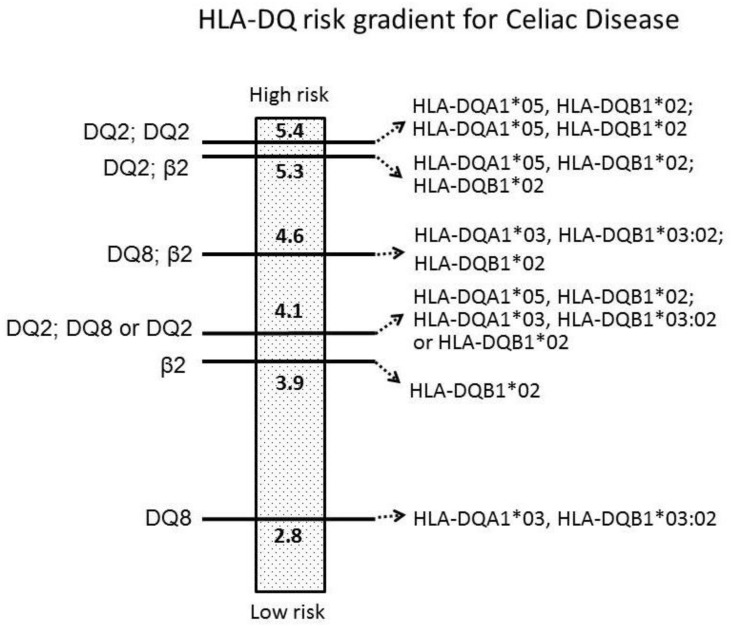
HLA-DQ risk gradient for Celiac Disease according to the odds-ratio (OR) values from our previous meta-analysis (modified from De Silvestri et al.).

It is well known that HLA-DQ genotyping is useful to ascertain the susceptibility to CD with very high—if not absolute—discriminatory power. Indeed, it is very unlikely that individuals who do not carry any specific HLA-DQ alleles, coding MHC-DQ2 (HLA-DQA1*05 + HLA-DQB1*02) and MHC-DQ8 (HLA-DQA1*03 + HLA-DQB1*03:02) heterodimers, can develop CD [1,8]. Such a knowledge resulted to be very useful in the diagnostic approach to some complex cases (e.g. patients with antibody deficiencies) and, importantly, was able to avoid the duodenal biopsies in those children fulfilling some specific clinical and serological criteria, according to the ESPGHAN (European Society for Pediatric Gastroenterology, Hepatology and Nutrition) guidelines, published in 2012 [9]. Recently, several groups started to investigate the possibility to take advantage of specific HLA-DQ genetic analyses for a potential multi-step approach to extend the screening for CD to children who are not considered to be at higher risk, as defined above. That may be feasible through a reduction of the costs for the genetic analysis, compared to the high-resolution HLA genotyping. For this purpose, one contributing factor may be limiting the genetic analysis to specific CD-predisposed HLA-DQ alleles and, in particular, to the HLA-DQB1*02 allele, which plays a relevant role in CD genetic predisposition, according to the risk gradient showed in Figure 1 [8,10,11,12]. Through this meta-analysis, we aim at providing further epidemiological support to this potential approach.

## 2. Materials and Methods

### 2.1. Protocol

**Figure 2:**
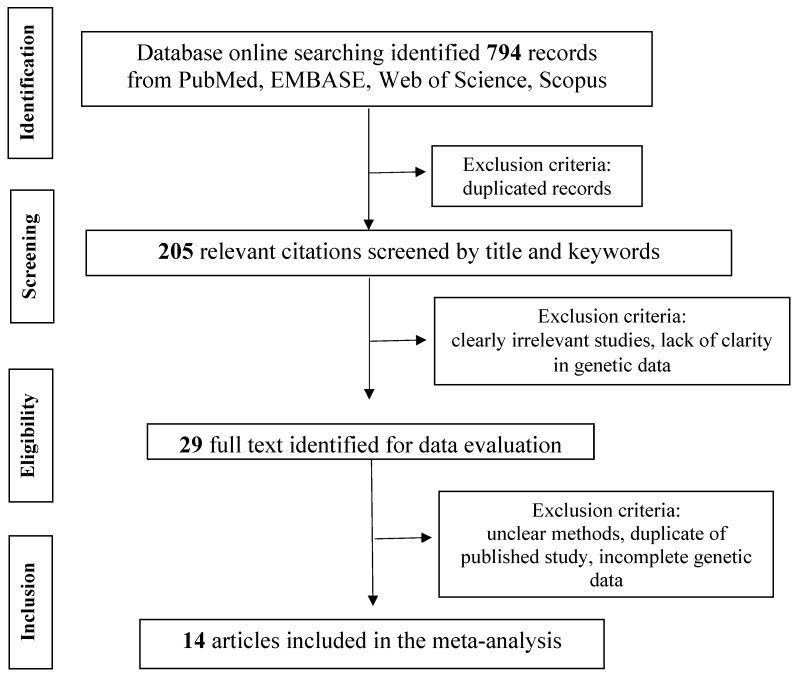
Flow diagram of the study following the PRISMA guidelines.

This work was written according to PRISMA guidelines [13], as described in Figure 2. Through this meta-analysis, we aimed at quantitatively evaluating the association between HLA-DQ polymorphisms and the susceptibility to CD in FDRs of pediatric CD patients.

### 2.2. Search Strategy

We performed a search of PubMed, EMBASE, Web of Science, and Scopus, retrieving all publications (case-control study, cross-sectional, and retrospective cohort study) on the association between HLA class II polymorphisms and first-degree relatives (FDRs) of CD children. We searched all articles published up to September 2018 in several languages (English, French, German, Italian, Portuguese, and Spanish).

We performed the search strategy using a free-text search (keywords) and thesaurus descriptors search (MeSH and Emtree) for each concept, adapted by a trained librarian for all the selected databases. In detail, an expert librarian performed the search by using the following terms: (“celiac disease” [MeSH] OR “celiac disease” [tiab] OR “coeliac disease” [tiab]) AND (“Histocompatibility Antigens Class II” [Mesh] OR “Histocompatibility Antigens Class II” [tiab]) AND (“nuclear family” [mesh] OR relative* [tiab] OR sibling* [tiab] OR parent* [tiab]) AND (“mass screening” [mesh] OR screening [tiab] OR prevalence[tiab] OR “Prevalence” [Mesh] OR “Predictive Value of Tests” [Mesh] OR “predictive value” [tiab]). In Embase, the search used the following terms: (‘celiac disease’/exp OR ‘celiac disease’:ti,ab OR ‘coeliac disease’:ti,ab) AND (‘HLA antigen class 2’/exp OR “Histocompatibility Antigens Class II”:ti,ab) AND (‘nuclear family’/exp OR relative*:ti,ab OR sibling*:ti,ab OR parent*:ti,ab) AND (‘screening’/exp OR screening:ti,ab OR ‘Prevalence’/exp OR prevalence:ti,ab OR ‘predictive value’/exp OR ‘predictive value’:ti,ab). In Web of Science, the search used the following terms: (“celiac disease” OR “coeliac disease”) AND HLA AND (relative* OR sibling* OR parent*) AND (screening OR prevalence OR “predictive value”). In Scopus, the search used the following terms: “celiac disease” OR “coeliac disease” AND HLA AND (relative* OR sibling* OR parent*) AND (screening OR prevalence OR “predictive value”).

### 2.3. Data Extraction

After a critical reading of the articles, two investigators independently performed data extraction according to the following inclusion criteria: HLA class II genes, any DQ and DR molecules, and celiac disease diagnosed following the clinical criteria set by Meeuwisse (1969–1970), Walker-Smith et al. (1990–2012), and Husby et al. (2012 ESPGHAN guidelines) [9,14,15]. The third participant was consulted for discussion to reach an agreement concerning discrepancies.

### 2.4. Data Synthesis and Meta-Analysis

STATA 14.2 (StataCorp., College Station, TX, USA) and METADISC 1.4 were used for statistical analysis to perform meta-analysis the [16]. Heterogeneity was checked through the χ^2^-test and the I-squared statistics [17]. The criteria for identification of heterogeneity were *p* values less than 0.10 for the χ^2^-test and an I-squared value greater than 50%. When there was no statistical evidence for heterogeneity in effect sizes, we used the fixed-effect model to analyze odds ratios (ORs) or relative risks in FDRs. When significant heterogeneity was identified, we used the random-effects model (REM) and explored sources of significant heterogeneity [18,19].

We considered all studies including subjects analyzed for both the HLA-DQA1*05 and HLA-DQB1*02 alleles (coding the DQ2 molecule) and/or for the HLA-DQA1*03 and HLA-DQB1*03:02 alleles (coding the DQ8 molecule). For each selected study, we calculated sensitivity, specificity, positive likelihood ratio (LR+), and negative likelihood ratio (LR–) to develop CD. In order to produce clinically useful statistics, we calculated the pooled LR+ and LR– values. For all estimated values, we provided the 95% confidence interval (CI).

## 3. Results

### 3.1. Study Selection 

Our search strategy yielded 794 papers for consideration. Following elimination of duplicates, 205 titles and/or abstracts were reviewed. Of these, 176 were excluded and, among the remaining 29 full-text manuscripts, 14 studies were deemed eligible for inclusion and were submitted to data extraction and analysis (refer to Figure 2). These studies were published between 1999 and 2016 and, collectively, enrolled 3063 FDRs. In detail, our analysis included three studies from India, two studies each from Chile, Italy, and Spain, and 1 study each from Brazil, Cuba, Jordan, Finland, and the USA [20,21,22,23,24,25,26,27,28,29,30,31,32,33].

Among the 3063 FDRs, 1720 patients were MHC-DQ2 or -DQ8 carriers, but only 352 were diagnosed with CD; among these, 337 patients were MHC-DQ2 or -DQ8 carriers. Thus, the prevalence of CD among FDRs was around 11.5% (CI 10–12).

### 3.2. Study Quality

The quality of selected studies, in terms of laboratory methods, methods description, statistical methodology and clinical features, was assessed according to PRISMA standards and resulted to be appropriate.

### 3.3. Meta-Analysis According to the Complete MHC-DQ2 and/or DQ8 Genotype

In our meta-analysis, we expressed these parameters as positive and negative likelihood ratios (LR+ and LR−, respectively) for CD, according to the presence of the HLA-DQ genotype coding complete MHC-DQ2 and/or MHC-DQ8 molecules. The pooled estimation of LR+ was 1.449 (CI 1.279–1.642), whereas LR− was 0.187 (0.096–0.362). While this HLA-DQ background is known to provide a low specificity (Table 1), its presence is actually characterized with very high sensitivity for CD, showing a good discriminatory power between genetically predisposed CD (not necessarily affected) patients and those who will not develop CD, as shown in Table 2.

### 3.4. Meta-Analysis According to the Isolated Presence of HLA-DQB1*02 Allele

DQB1*02 sensitivity was 0.938 (CI 0.891–0.968) and specificity was 0.425 (CI 0.400–0.451). We meta-analyzed the LR+ and LR− of FDRs according to the presence of the DQB1*02 allele. The pooled estimate of LR+ was 1.659 (CI 1.302–2.155) (Table 3), whereas LR− showed a good discriminatory power of 0.195 (CI 0.068–0.558) (Table 4).

## 4. Discussion

This meta-analysis confirmed the very high negative predictive value associated with the absence of the HLA-DQ genotype, coding MHC-DQ2 and/or -DQ8, as regards the risk to developing CD. This long-established knowledge has been exploited to complete and/or refine the diagnostic work-up of patients suspected to be affected with CD, but having doubtful histopathological findings or concomitant diseases that can impair the reliability of the serological screening (e.g., IgA deficiency, common variable immunodeficiency) [22,34]. More recently, this genetic analysis has been included in the ESPGHAN guidelines to diagnose CD without performing any duodenal biopsy in children with consistent symptoms, high-titer of anti-tTG IgA, and EMA positivity [9]. However, beyond these practical conditions, the poor positive predictive value of being carrier of MHC-DQ2 and/or -DQ8 heterodimers, cannot provide any additional usefulness to the diagnosis of CD, in addition to confirming the necessary genetic predisposition.

Additionally, in this meta-analysis we separately analyzed the positive and negative predictive values (expressed as positive and negative LRs, respectively) related to the presence and absence of the HLA-DQB1*02 allele. Recently, Megiorni et al. reviewed the role of HLA-DQA1 and HLA-DQB1 in the predisposition to CD. They described a risk gradient whereby patients who are DQ2/DQ8 heterozygous and DQ2 homozygous showed a very high risk and, to follow, there were patients who were DQ8 homozygous, DQ8 heterozygous, along with DQ2 heterozygous, and, then, people carrying a double dose of DQB1*02 only [35]. Importantly, these latter patients showed a similar risk of CD as the previous categories, although they did not carry the complete MHC-DQ2 or MHC-DQ8 heterodimer.

Recently, a previous meta-analysis by our group supported this observation: we showed that a double dose of HLA-DQB1*02 was associated with the highest risk to develop pediatric CD (OR > 5), regardless of other HLA-DQ alleles. Moreover, even a single “dose” of HLA-DQB1*02 was associated with a relatively high risk (OR around 4) for pediatric CD. Basically, our statistical analysis suggested that children carrying only one HLA-DQB1*02 copy (without any other allele related to MHC-DQ2 or MHC-DQ8 molecules) have a similar predisposition/risk to become celiac as children expressing the full MHC-DQ2 and/or MHC-DQ8 molecules [8]. Accordingly, the original research by Megiorni et al., including 437 Italian children with CD and 551 controls, described a disease risk of 1:26 for children being homozygous for HLA-DQB1*02 (despite the absence of the other genes coding for DQ2 or DQ8); children being double heterozygous DQ2/DQ8, heterozygous DQ2 with double dose HLA-DQB1*02, and DQ8 heterozygous along with one HLA-DQB1*02 allele, showed a disease risk of 1:7, 1:10, and 1:24, respectively [28]. Therefore, all these studies suggested a major relevance of HLA-DQB1*02 allele in conferring the risk to develop pediatric CD, rather than the expression of the full MHC-DQ2 and/or -DQ8 heterodimers. Moreover, a risk gradient according to the dose (“single” or “double” copy of HLA-DQB1*02) has been evidenced.

Through our differential meta-analysis, comparing the presence of the full MHC-DQ2 and/or DQ8 genotypes and the isolated presence of HLA-DQB1*02 allelic variant, we found that the negative LR (namely the negative predictive value) was basically the same (0.187 vs. 0.195). Unfortunately, we could not obtain enough data to perform the same statistical analysis considering the HLA-DQB1*03:02 solely, as its frequency in the general population and CD patients is much lower compared to the HLA-DQB1*02 allele.

However, some molecular studies supported this concept that HLA-DQB1*02 may play a major role in the interaction between class II MHC molecule and the gliadin-derived peptide to be presented to T-lymphocytes, in order to trigger all immunological events involved in the pathogenesis of CD [36]. Indeed, the high content of proline and glutamine residues of MHC-DQ2-restricted gliadin epitopes resulted to be fundamental for the interaction and binding to the class II MHC molecules. One research showed that some specific DQ2 β chain residues, participating in the formation of the peptide-binding cleft (particularly Arg-β70 and Lys-β71 of β chain encoded by HLA-DQB1*02), are mainly responsible for the interaction with several residues of the gliadin epitope and, thus, may be critical to the CD predisposition [37].

Previously, we showed that 90–95% of CD children seem to carry at least a single copy of HLA-DQB1*02, regardless of the remaining HLA-DQ genotype [8]. Moreover, we supported this finding in our monocentric case series, including 269 children with CD, where >97% of all these CD children possessed at least one copy of HLA-DQB1*02 allele in their individual genotype [38]. Here, we looked at the HLA-DQ asset in the FDRs of pediatric CD patients and we found that the almost absolute negative LR was maintained, even when we considered only HLA-DQB1*02 in our analysis. This comparison suggests that the absence of this allele from the individual HLA-DQ genotype might rule out any individual predisposition to develop CD, as well as the analysis of the full genotype coding complete MHC-DQ2/DQ8 heterodimer(s) can do, without any significant decrease in the negative predictive value for CD.

These observations may contribute to the debate about the potential and cost-effective implementation of wider or mass-screening strategies for CD in children. Indeed, a low-cost HLA-DQ analysis, specific for CD predisposition, may allow to select those 30–40% of children who really deserve the serological screening [39,40,41].

The qualitative analysis to screen the presence of HLA-DQB1*02 in order to establish the genetic predisposition in the general population, may be one potential approach, and the complete HLA-DQ genotyping might be reserved to children with clinical suspicion, if needed [8,10,12]. Of course, further epidemiological, clinical and genetic researches are required in order to establish if this approach may be appropriate, feasible and cost-effective. However, other researchers considered a potential multi-step approach to screen CD, starting from the analysis of the specific genetic predisposition to CD through low-cost molecular methods. Recently, Verma et al. proposed a rapid HLA-DQ typing method to identify subjects genetically susceptible to CD. Basically, they performed a PCR through a kit containing the primers for the HLA-DQ target alleles only, on blood samples from CD patients, FDRs and controls. They could show an excellent concordance with the results obtained through conventional high-resolution HLA-DQ typing, in terms of presence or absence of HLA-DQ2 and HLA-DQ8 alleles [11]. The implementation of cost-effective screening strategies may be very helpful, not only in Western countries (where CD has been widely studied and described), but also in developing countries, where a number of health system-related barriers have not permitted an evidence-based approach to the diagnosis of CD, yet [42,43,44].

Therefore, if our observations will be supported by further and independent studies, those may represent an additional contribution to reduce the cost of a targeted genetic analysis for CD.

## 5. Conclusions

Through a differential meta-analysis, comparing the presence of the genotype coding the full MHC-DQ2 and/or DQ8 molecules and the isolated presence of HLA-DQB1*02 allelic variant, we found that the LR− of the latter analysis maintained the same value. This observation, along with the previous evidences, might be useful to consider potential cost-effective widened screening strategies for CD in children.

## Figures and Tables

**Table 1:** Positive likelihood ratio (LR+) of MHC-DQ2 and/or -DQ8 genotype in first-degree relatives (FDRs) of pediatric celiac disease (CD) patients (Heterogeneity χ^2^ = 132.54 (d.f. = 13), *p* < 0.001; Inconsistency (I-squared) = 90.2%; and estimate of between-study variance (Tau-squared) = 0.0455).

Summary—Positive Likelihood Ratio (Random Effects Model)				
Study (Year)	Country	LR+	95% Conf. Interval	% Weight
Araya et al. (2015)	Chile	1.008	0.749–1.357	5.92
Araya et al. (2000)	Chile	7.557	4.475–12.762	3.47
Bonamico et al. (2006)	Italy	1.779	1.572–2.014	8.20
Cintado et al. (2006)	Cuba	1.054	0.809–1.372	6.38
Elakawi et al. (2010)	Jordan	1.487	1.061–2.084	5.40
Farre et al. (1999)	Spain	1.563	1.317–1.854	7.64
Karinen et al. (2006)	Finland		1.008–1.255	8.35
Martins et al. (2010)	Brazil	1.691	1.393–2.052	7.35
Megiorni et al. (2009)	Italy	1.707	1.590–1.832	8.67
Mishra et al. (2016)	India	1.304	1.126–1.511	7.94
Rubio-Tapia et al. (2010)	USA	1.413	1.302–1.533	8.59
Singla et al. (2016)	India	1.148	1.040–1.268	8.45
Srivastava et al. (2010)	India	1.063	0.783–1.443	5.82
Vaquero et al. (2014)	Spain	1.530	1.309–1.789	7.83
(REM) pooled LR+		1.449	1.279–1.642	

**Table 2:** Negative likelihood ratio (LR−) of MHC-DQ2 and/or -DQ8 genotype in FDRs of pediatric CD patients (Heterogeneity χ^2^ = 20.34 (d.f. = 13), *p* = 0.087; inconsistency (I-squared) = 36.1%; and estimate of between-study variance (Tau-squared) = 0.4949).

Summary—Negative Likelihood Ratio (Random Effects Model)				
Study (Year)	Country	LR−	95% Conf. Interval	% Weight
Araya et al. (2015)	Chile	0.933	0.065–13.461	4.86
Araya et al. (2000)	Chile	0.081	0.006–1.178	4.86
Bonamico et al. (2006)	Italy	0.107	0.028–0.416	11.74
Cintado et al. (2006)	Cuba	0.641	0.041–10.017	4.64
Elakawi et al. (2010)	Jordan	0.217	0.015–3.170	4.83
Farre et al. (1999)	Spain	0.108	0.007–1.636	4.72
Karinen et al. (2006)	Finland	0.526	0.265–1.044	18.52
Martins et al. (2010)	Brazil	0.158	0.024–1.054	7.99
Megiorni et al. (2009)	Italy	0.044	0.006–0.310	7.73
Mishra et al. (2016)	India	0.188	0.047–0.757	11.43
Rubio-Tapia et al. (2010)	USA	0.042	0.003–0.656	4.61
Singla et al. (2016)	India	0.173	0.011–2.730	4.61
Srivastava et al. (2010)	India	0.652	0.045–9.460	4.85
Vaquero et al. (2014)	Spain	0.043	0.003–0.674	4.60
(REM) pooled LR−		0.187	0.096–0.362	

**Table 3:** Positive likelihood ratio (LR+) related to the DQB1*02 allele in FDRs of pediatric CD patients (Heterogeneity χ^2^ = 89.02 (d.f. = 6), *p* < 0.001; inconsistency (I-squared) = 93.3%; and estimate of between-study variance (Tau-squared) = 0.0906).

Summary—Positive Likelihood Ratio (Random Effects Model)			
Study (Year)	LR+	95% Conf. Interval	% Weight
Araya et al. (2015)	7.557	4.475–12.762	9.44
Cintado et al. (2006)	1.054	0.809–1.372	14.07
Elakawi et al. (2010)	1.487	1.061–2.084	12.72
Farre et al. (1999)	1.563	1.317–1.854	15.58
Karinen et al. (2006)	1.125	1.008–1.255	16.32
Martins et al. (2010)	1.691	1.393–2.052	15.24
Megiorni et al. (2009)	1.707	1.590–1.832	16.64
(REM) pooled LR+	1.659	1.302–2.115	

**Table 4:** Negative likelihood ratio (LR−) related to the DQB1*02 allele in FDRs of pediatric CD patients (Heterogeneity χ^2^ = 12.06 (d.f. = 6), *p* = 0.061; inconsistency (I-squared) = 50.2%; and estimate of between-study variance (Tau-squared) = 0.9053).

Summary—Negative Likelihood Ratio (Random Effects Model)			
Study (year)	LR−	95% Conf. Interval	% Weight
Araya et al. (2015)	0.081	0.006–1.178	10.43
Cintado et al. (2006)	0.641	0.041–10.017	10.03
Elakawi et al. (2010)	0.217	0.015–3.170	10.38
Farre et al. (1999)	0.108	0.007–1.636	10.18
Karinen et al. (2006)	0.526	0.265–1.044	28.05
Martins et al. (2010)	0.158	0.024–1.054	15.66
Megiorni et al. (2009)	0.044	0.006–0.310	15.26
(REM) pooled LR−	0.195	0.068–0.558	
